# The Conceptual and Practical Ethical Dilemmas of Using Health Discussion Board Posts as Research Data

**DOI:** 10.2196/jmir.2435

**Published:** 2013-06-07

**Authors:** Carol S Bond, Osman Hassan Ahmed, Martin Hind, Bronwen Thomas, Jaqui Hewitt-Taylor

**Affiliations:** ^1^School of Health and Social CareBournemouth UniversityBournemouthUnited Kingdom; ^2^Media SchoolBournemouth UniversityBournemouthUnited Kingdom

**Keywords:** social media, research ethics, health, research design

## Abstract

**Background:**

Increasing numbers of people living with a long-term health condition are putting personal health information online, including on discussion boards. Many discussion boards contain material of potential use to researchers; however, it is unclear how this information can and should be used by researchers. To date there has been no evaluation of the views of those individuals sharing health information online regarding the use of their shared information for research purposes.

**Objective:**

To explore the views of contributors to online diabetes discussion boards with regards to if (and how) they feel their contributions to boards should be used by health researchers.

**Methods:**

A qualitative approach was employed using online semistructured asynchronous (email) interviews. Interpretative description methodology was used to assess the interview transcripts, and quotations were extracted and anonymized to support each theme.

**Results:**

26 interviews were carried out. Participants agreed that forum posts are in the public domain and that aggregated information could be freely used by researchers. This was agreed to be a good way of ensuring that the view of people living with diabetes is being heard in research. There was no consensus on the need for permission to use individual information, such as quotations, with some people happy for this to be freely used and others feeling that permission is necessary.

**Conclusions:**

Participants acknowledged the dichotomy of having placed information into the public domain in an unrestricted way, with some interviewees also wanting to retain control of its use. The Internet is a new research location, and rather than trying to apply traditional ethical norms to this new genre, a new modus operandi is required. The authors propose introducing new norms for presenting research carried out with online discussion boards.

## Introduction

The widespread use of the Internet throughout modern society has led to individuals being able to connect with others with the same health condition as them on a global scale [1]. Without rehearsing a comprehensive history of the Internet, it has progressed from a relatively closed community of people who needed some technical knowledge or skills to used shared spaces such as Bulletin Boards and MUDs (multi-user domains), to an ubiquitous user-friendly network with social spaces characterized by easy-to-use discussion boards and social media sites. This permeation has significantly affected the nature and extent of the user base, who now need little knowledge and few skills. One of the first attempts to measure Internet and Web use was carried out in 1996 [2], and although there was debate about the accuracy of extrapolating the survey data to the population of North America, final figures were agreed to be approximately 19.4 million Internet users and 14.6 million Web users. This represented approximately 7% of the population. By 2012, this had risen to around 88% of the population [3].

This change can also be seen in the health arena. Websites provide information and advice for most long-term health conditions [4-6], and many of these contain discussion boards where people can share experiences and support each other [7-9]. The evolution of the Internet from a limited, technical resource to today’s dynamic “Web 2.0” where people are able to share information means that increasing numbers of people living with a long-term condition are now putting personal health information into the public domain, including on discussion boards [10].

Online discussion boards contain material that is potentially of use and of interest to researchers, especially to researchers interested in the experiences of those living with a long-term condition. Issues such as self-management, concordance with medicine and other regimes, and interactions with health care professionals are all discussed freely in these forums, with hundreds and sometimes thousands of individuals contributing their opinions on a certain topic. However, in keeping with studies conducted on any cohort, researchers must adhere to the appropriate ethical guidelines when conducting their research [11].

One of the early attempts to establish some ground rules for ethical online research was made in 2002 by the Association of Internet Researchers (AoIR) [12]. Since this research was undertaken, these recommendations have been updated [13]. The report authors acknowledge that much has changed in the field of Internet studies over the period between the versions, including a multitude of devices and the “interweaving of online and offline activities and experiences” (p. 2). These guidelines address the challenge of trying to apply the concept of a “human subject” to an online environment but are generic in discipline terms and do not specifically address health research.

The European Commission’s Information Society Technologies (IST) Programme funded the RESPECT project [14], which drew up professional and ethical guidelines for carrying out socioeconomic research. The RESPECT guidelines reinforced the methodological challenges associated with online research (identifying dangers of conducting research in this manner) but stopped short of giving specific information tailored to the needs of online communities.

In the United Kingdom, the Department of Health’s Research Governance Framework for Health and Social Care has set out the expectations for those studying NHS (National Health Service) patients, their carers, and significant others [15]. The overarching expectation is that “The dignity, rights, safety and wellbeing of participants must be the primary consideration in any research study” [15]. However, this framework does not include specific mention of how these principles are best applied in the online environment.

Sharing health information online has brought about new opportunities for researchers and health professionals, providing a repository of information that has the potential to help clinicians better understand the needs associated with specific clinical populations. It has been suggested that health researchers have been slow to seize the opportunities of online research in comparison to disciplines such as media studies [16] and that ethical issues are partly responsible for this. This has been an ongoing debate. In 2000, two researchers [17] published an exploration of the dilemmas they faced in trying to carry out research within a list serve-based community. In 2007, Whitehead [18] found that the research community was divided about the correct approach to take when considering the ethical issues, and in 2009, Holmes [19] was of the opinion that the ethical standards for Internet research were not well developed. The discourse relating to the ethical use of information shared online has centered upon the views and experiences of researchers [20-22] and has not been focused exclusively on health information.

One of the first discussions about the ethics of health research online was in 2001 [23], and although this was before Web 2.0, the challenges identified are still relevant, as is the conclusion that best practice guidelines are needed. The issue of how ethical principles can be applied to online health research has provided a challenge to researchers [24]. Much of the work undertaken to date, such as the AoIR guidelines [13], has been developed in consultation with researchers.

To date, the views of those posting health information online in relation to how they anticipate the information they post being used has not featured significantly in the debate. Eysenbach and Till [23] reviewed comments posted on health-related discussion boards in 2001, concluding that members of Internet communities do not expect the posts they make to be used by researchers. This research identified the issue of the blurring of public and private spaces when using health-related discussion boards. Since that study, there has been a rapid growth in online interactions for health-related purposes [25], with recent figures suggesting up to 80% of Internet users have searched for health information online [26]. The emergence of social networking sites has had a huge impact on how individuals communicate and share health information online. The largest social networking site, Facebook, which was launched only in 2004, currently has more than 1 billion active users [27] and its use for health-related purposes has been reported for many conditions [28-30]. The extent to which individuals across the world are now using online spaces to share health information was unimaginable when questions relating to ethical issues in using online places for research were first identified.

In order for researchers to be able to understand what constitutes the ethical use of online health information, it is important to address the lack of evaluation of the views of those posting health information online. This study seeks to bring their voices into the knowledge base by examining the views of people living with diabetes who share health information on online discussion boards. The goal of this study was to undertake semistructured interviews with users of online diabetes discussion boards, in order to better understand their views towards how the information that they share in these forums is used by researchers.

## Methods

### Ethical Approval

Ethical approval for this study was granted by the Ethics Committee of the School of Health and Social Care, Bournemouth University [31]. Data collection commenced in April 2012 and concluded in May 2012.

### Study Design and Participants

The study used online semistructured asynchronous (email) interviews [32]. Both CSB, the primary investigator (PI) and the project researcher, OHA, had experience in online interviewing techniques. The target number of 24 participants was set prior to recruitment, with the ability to increase this should the need arise. Deciding how many interviews to conduct for qualitative research is an inexact science and very dependent on the context [33]. Drawing on previous experience, it was correctly anticipated that saturation of data would be reached by this stage.

### Identification of Participants

**Figure 1:**
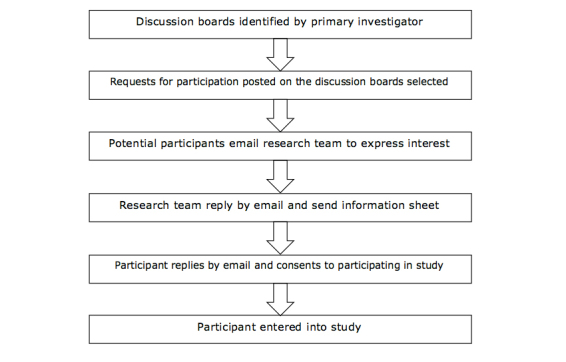
Participant recruitment and consent process.

Figure 1 outlines how participants were identified and recruited into the study. Four active diabetes forums were identified by the PI and chosen to recruit participants into the study. Four forums were selected to ensure that any particular character or interests on a board would have undue influence on the findings. None of these forums required membership in order to read the posts; however, two of the groups did require membership in order to post on the forum. Moderators and administrators of the forum were approached prior to posting on the forums in order to gain their approval for posting for research purposes. These moderators and administrators were also asked if they were willing to participate in the study themselves. This led to participants having a range of posting experience encompassing community leaders to occasional contributors.

### Inclusion/Exclusion Criteria

Participants were eligible for inclusion in the study if they were an active member on one of the forums identified in the study. Active was defined as having made at least one post. There were no exclusions other than, as the interviews were conducted in English, people who were not sufficiently fluent in written English were unable to participate.

### Recruitment

CSB and OHA posted in each of the forums on different threads within the forum in order to give the recruitment posts more publicity. The recruitment posts described the study and provided our contact details. Individuals interested in participating were invited to contact us by email for more information. Once interested individuals had made contact with the research team, they were sent a brief overview of the study along with an information sheet. Following this, if the participants were happy to participate, they were sent the first interview questions by email and the interview was started.

### Data Collection

The data from participants were collected using asynchronous semistructured interviews [32]. The semistructured nature of these interviews meant that similarly themed information was collected from the participants, while the fact the interviews were asynchronous enabled participants to respond to questions in their own time (but within the time constraints of the research program).

Participants were sent several emails during the course of the interview with each email containing 1-2 questions. The questions (see Multimedia Appendix 1) aimed to ascertain whether participants felt it was acceptable for researchers to use information on health discussion boards, what permission should be sought prior to using this information, and whether the length of time since the post was made influenced the need to obtain permission. Once the participants had answered all of the questions, they were thanked for their contributions and their participation in the study was over.

### Data Analysis

**Figure 2:**
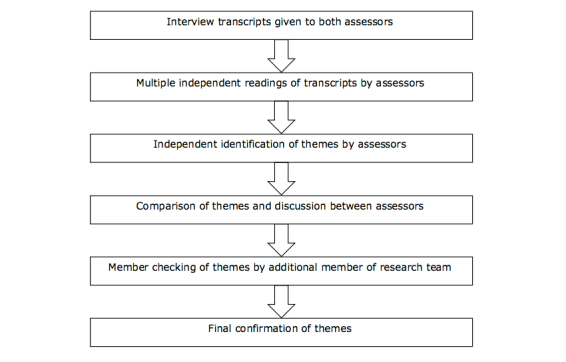
Data analysis process.

A qualitative approach using interpretative description [28] was employed to assess the responses to the semistructured interviews. An inductive approach was adopted, allowing the themes to emerge from the data rather than testing previously identified themes on the data. The rigor of this analysis was based on Guba and Lincoln’s principles of credibility confirmability and dependability [34,35]. This was achieved through prolonged engagement with sufficient depth of data; peer debriefing and analysis of materials; linking assertions, findings, and interpretations with the data and third party auditing of the data collection and analysis process. The process is set out in a flow chart (Figure 2) highlighting the stages in the process of analysis. CSB and OHA read each transcript several times independently and loosely attributed themes to the data with no consultation occurring between the assessors at this stage. Discussion was then undertaken to compare the themes identified and to resolve any areas of disagreement with theme allocation. Quotations were extracted to support themes, and each participant was assigned a number (eg, P04) when using the supporting quotations. Following this process and the identification of themes, their findings were sent to the other members of the research team (BT and MH) in order to verify the themes. In view of the lack of consensus on some issues among the study participants, a final check was carried out by someone not previously involved in the analysis (JHT) who reviewed the themes and conclusions to ensure consistency.

## Results

Following the requests for recruitment posted on the discussion boards, 33 individuals expressed an interest in participation and were contacted by the research team. After this initial contact, 30 participants consented and responded to at least one question in the interview sequence, while four participants who consented did not complete the interview questions and did not respond to further follow-up prompts. The remaining 26 participants completed all of the semistructured interview questions and their answers were included for analysis.

It was anticipated that each of the interviews would be completed in a short space of time, and all of the interviews were completed in less than 10 days. Of the 26 completed interviews, 12 participants were identified (from their username/email correspondence) as being male, 9 as being female, while it was not possible to classify the remaining 6 participants from their username/email correspondence.

Following the analysis of the interview transcripts, several key themes emerged.

### The Views of People Living With Diabetes Are Needed

There was general support for people using information posted on discussion boards in research. The need for people living with diabetes to have a voice was clearly felt by respondents: “it’s nice to see that someone takes note of what we, the diabetics have to say” (P23) and

I think that this type of information is vital to any type of research. Patients opinions are often not “heard” because of time constraints etc., these forums give an insight into what we feel we need from our HCPs and just as importantly…what we don’t need!

P26

I have no reservations about your mining information from forums…it will provide much information about the human side of illness and how individuals singly and collectively approach and cope through sharing. Dare I say its importance cannot be understated.

P27

There was also an altruistic view that this use should be for the “general good”: “presuming the research is for the general good and benefit of many” (P12) and

I think I’m probably happy for you to use direct quotes from my comments without permission, providing it is for research purposes and not for commercial gain.

P4

### Posts Made Are in the Public Domain

It was generally accepted by participants in this study that once information is posted on a forum, it is available to the public and thus could be accessed and used for research purposes: “Simple – it’s the Internet! Everything posted on the site is automatically in the public domain and available for anyone to read” (P15).

Some people acknowledged that the nature of online communities could create a different impression: “As a poster you really only think you’re saying things to the forum community but of course you are saying it to a far wider audience” (P29).

Some respondents struggled with the dichotomy in their responses:

As someone who uses an Internet forum, on the one hand I’m happy for my information to be available publicly and for free to anyone who might need it, and on the other hand I wouldn’t appreciate that information contributing to something beyond “personal use” without my knowledge. Although at the same time, like any author, I have to be aware that that almost certainly will happen and I really have no control over what I’ve written once it’s out there for anyone to see.

P16

Having acknowledged that they were putting information into the public domain, two more related themes emerged. Some people felt it could be used unreservedly, while others still felt there should be restrictions.

“The information is in the public domain, AND I have no problems with it being used” emerged as a theme: “When people post on the Internet, it is there for all to see. They should not complain if it can be harvested and used for the general good” (P12), “As they (forums) are open to the public and in the public domain why would it not be OK to use the information?” (P19), “I think information obtained from a discussion board when anonimised should be usable in your research, as it has been published on a public area” (P9), and “As the information is posted on the very public Internet, I don't think there is a need for permission to use the posts”. (P10)

The difference between boards that are open to all to read, and those that require membership to access was mentioned by one respondent:

I feel if a forum is viewable to the public, IE you don’t have to be a member to view any of the forum threads, then you or anyone else can use any of the information you find on any forum.

P20

“The information is in the public domain, but I’d be uncomfortable about it being freely used” also emerged. Some respondents were clearly struggling with views they felt to be contradictory, on the one hand acknowledging that their posts were in the public domain, but also feeling ongoing ownership of them:

I write a blog about my experience of diabetes and would feel very aggrieved if I found any of it quoted in a medical research paper without having been asked. I realise this is slightly contrary (since I am posting and effectively actively encouraging readership) but nevertheless it would feel like “theft” of my content.

P17

Even though posts made on a public domain are, well, public I think it is a common courtesy to approach the poster by private message if necessary to ask their opinion.

P26

Legally the comments are in the public domain and uncopyrighted so they are “free” for anyone to use. Morally, I think that permission should probably be requested first to repeat comments verbatim, even rephrased one should probably state source.

P9

### Permission

Respondents were asked if they thought researchers need permission to use information in their posts. Responses grouped into two subthemes.

#### Permission Not Needed

Those who felt their information was in the public domain and therefore available to be used did not feel there was any need for permission to be sought:

If the forum is viewable to the public, then no permission is necessary.

P20

If the forum is viewable to the public, then no permission is necessary.

P20

Obviously u won't be using anyone's name so I don't think u need to obtain any permission.

P25

When people post on the Internet, it is there for all to see. They should not complain if it can be harvested and used for the general good.

P12

I think I'm probably happy for you to use direct quotes from my comments without permission, providing it is for research purposes and not for commercial gain.

P4

#### Permission Required in Some Circumstances

Those who acknowledged the public nature of the forum but expressed reservations felt that some sort of permission for use should be obtained: “I think informed consent should be obtained from the person who posted the information” (P33) and “Using posts which were made in ignorance of them being used in the way you intend does not sit easily with me. I would hope that their use would at least be with the permission of the site administration” (P32).

There were however divergent views and no consensus over the role of site administrators in giving permission: “Absolutely not, what right do they have to share what is on the forum they admin” (P10) and “I think it would be a complete betrayal if (admin) were to give permission on behalf of the members” (P3).

The use of quotations was one area where this group people tended to agree:

If you took a quote direct from what someone has said “eg when I changed to a low carb diet I lost 2 stone and my Hba1c dropped to...” then I think you do need to gain permission from the poster.

P3

If there were ever any intention to use or quote a specific post or case study then I would feel it would be absolutely essential to get approval from the individual before going ahead.

P17

A different view was taken to “aggregated data”: “if you’re using the data in some kind of statistical analysis – and not quoting directly the posting then I’d say no permission is probably needed” (P10) and

If it’s an “abstract” I don’t think you need permission. For example if you discover from reading forums that many HCPs give dietary information to diabetics which is plainly making their condition worse, and that many diabetics find that eating a diet which is low in carbohydrates is more beneficial, then you could “generalise” about the situation. 

P3

If the information you collect ends up being essentially anonymous numbers…charts or that kind of thing then I don’t think you need any individual’s permission. If you want to use actual quotes from people that's a different matter as even if you make the quote anonymous in your research.

P29

Provided no quotes are attributed I am OK with your extraction of any postings.

P27

One would expect information to be depersonalised either by general aggregation or by use of pseudonyms in specific cases.

P12

Few people would have a problem with generalised and anonymised references.

P17

The time that has passed since the post was made was not considered to be relevant in the need to obtain permission:

A post I made two years ago, even if I’ve forgotten about it, is no less mine and I would be no less upset to find it had been used without my knowledge than a post I made yesterday.

P16

### Use of Citations

There were divergent views about attributing information to the poster. Some people felt that if quotations were to be used the author and/or forum should be cited:

I think in my mind it’s a bit like any other written work, a book in a library for example. The text is there, for anybody to read and learn from, so in that sense it’s public. On the other hand if we want to quote that author in writing a review or a paper, we have to include reference information.

P16

If someone decided to republish my post in another forum or document, I would expect my comments to be kept in context and credited to me.

P04

Other respondents valued anonymity over credit: “(permission isn’t needed) As long as you don’t identify the poster by more than sex, age, and type of diabetic” (P20) and “Obviously u won’t be using anyone’s name so I don’t think u need to obtain any permission” (P25).

The challenge of using quotations and maintaining anonymity was raised by one respondent:

If you want to use actual quotes from people that’s a different matter as even if you make the quote anonymous in your research it will be quite easy to find the author simply by typing in key phrases into Google which will then give links back to [the forum].

P29

## Discussion

### Principal Findings

The findings demonstrate a general agreement that the view of people living with diabetes should be heard by researchers and that the information on these forums contains information that is valuable to researchers. Participants had a range of experience including community leaders and occasional posters; it is possible that their experience of using discussion boards was a factor in how they saw the issues; however, this study did not attempt to explore this. It could be useful to carry out a quantitative study to test these findings with a large number of participants to explore if experience of using discussion board, or the Internet generally, is an influencing factor. Most participants appreciated that they had put their information into the public domain.

Concepts from mass media studies help to offer insight in order to better understand the process of sharing information online in this manner. The public sphere was initially proposed as an area where public opinion is expressed through rational debate and discussion by Habermas [36]. This view was formed before the Internet (with all of its powerful capabilities) was created and thus the extent to which this view can be applied to online spaces is not clear. Blogs have been likened [37] to the “letters to the Editor” that are common in traditional print media. The concept of “texts in the public domain” has also been discussed as being a social process of talk and text [38], with the authors concluding that where communication happens in ways other than face to face and has multiple audiences, a “pragmatic framework” for researchers to work with it needs to be developed.

Beyond the initial agreement about the public nature of their posts by our participants, two divergent subthemes emerged. One group was happy that they had put their information into the public domain and that it could therefore be reused by researchers. This approach and outlook could grow with the increased use of social media by society. Twitter for example has a “retweet” button, enabling the rapid dissemination of information. Retweeting is one of the core functions of Twitter [39], and it is considered completely acceptable to do so without seeking any prior permission. Basset [40] stated that the Internet is a cultural publication route, more akin to mass media, such as newspapers than any other medium. This “mass media” approach by researchers may be feasible for Twitter (and arguably blogs) where the author is writing to an unknown audience, although this is not supported by participant P17 from this study. Whether this approach can (or indeed should) also be applied to discussion boards is questionable, however, since participants are “talking” to other board users rather than intentionally addressing their comments to a wider, unknown audience.

The second subgroup in this study wanted to keep ownership of their words. While they were happy for aggregated data to be reused, they felt differently about quotations or the use of the information that could be traced back to them. Rather than applying the attribution norms of mass media communications, their views fit more comfortably into a traditional health research ethics framework. Some people wanted to be able to give (or withhold) permission for the use of the information they had shared. Others wanted to know that their words would remain confidential and not be able to be traced back to them. Rather than focusing on the paradoxical nature of this stance, ways of meeting the wishes of this group (without negating the ability to use information from people who want to make it available) need to be explored.

Eysenbach and Till [23] recommended that consent should be obtained before using verbatim quotes, a view supported by the participants in this research. Seeking to gain permission to use posts from online discussion boards for research purposes is, however, likely to render the research difficult at best and unfeasible at worst. Posts often need to be considered in the context of the discussion, so if one poster was happy to give permission and wanted their views to be included, and another withheld permission, the conflict would need to be resolved and a decision made about whose wishes have primacy.

Reaching posters to seek permission is an additional dilemma. Board membership is often transient, contact details are not given or where they are they are out of date, and the way some boards are set up people have to have a posting history before they can be accessed or used. That creates another ethical dilemma to be resolved. Consent by proxy (through board administrators) generated mixed feelings from respondents (sometimes strongly expressed) so would not be an ethical resolution to this situation either. Failure to secure consent from sufficient posters could mean that the data available to use are not balanced nor representative of the boards. If the data are rendered unusable because of this then the wish of some posters that their contributions are used for greater good cannot be met. The outcome of this would be that a valuable research resource that could help develop concepts and care to ultimately benefit the wider community, which the boards seek to support, will be lost.

The AoIR [13] identifies that the concept of “the human subject” does not fit well with many online environments and that practical considerations such as harm, vulnerability and identification may be more important. Trying to apply rules that were created for a different context does not provide solutions to these dilemmas, leading us to the inevitable conclusion that new situations such as this require new rules. Having considered the dichotomy that both researchers and participants acknowledge, we propose that a new conceptual framework for online research is needed and that further work is required to ensure that all stakeholders in the process are included in the discussion. Key messages that have been drawn from our research are:

- Contributors appreciate the value of their boards to research that will ultimately benefit their communities and wish it to be used.
- Using aggregated data is acceptable to the community that created it.
- Using quotations ranges from being totally acceptable to totally unacceptable.

The Internet is not one entity, and different aspects may require different approaches. The information openly available on online discussion boards does not fit into a human subject approach to research ethics, and while viewing it as more akin to text, mass media norms do not fit well either. It is a specific type of text—“personal health text” that requires sensitive handling and the generation of a new set of research rules.

We propose that the use of non-verbatim quotations should be considered as an alternative to verbatim quotations. At the simplest level, using nonverbatim quotations could be through editing the words of a single author to an extent by which it cannot be located by a search engine. As search engines become increasingly sophisticated, this solution is not likely to be easy to achieve, and indeed in time, this may become impossible. An alternative that we believe is preferable is to introduce a new concept to research—aggregated quotations. By this, we propose bringing several quotations around a topic together to maintain the essence of meaning, but also rendering it impossible to identify any individual poster.

The use of quotations is a way of presenting research that is generally linked with qualitative research [23] where the aim is to provide a richly textured and comprehensive set of data, without any gaps, and with the full breadth of interpretations included [41,42]. The value of verbatim quotations has been identified [43] to provide evidence of what was said; to present full or more clear explanation of participants’ views; as a means of illustrating the codes and categories that have been developed from the data; to give depth to participant’s meanings, “giving participants a voice”, and to make research reports more readable. All of these aims can equally be achieved using thoughtfully constructed aggregated quotations.

The aim of the study was to identify what Internet users found acceptable rather than to explore the issue through a methodological lense. The findings, however, have implications for research methodology. It is beyond the scope of this paper to develop a detailed methodological exposition; however, rather than viewing this new approach as fitting into a qualitative framework, we see this proposal as being part of the formation of a new genre of research that focuses on the specific characteristics of Internet-based research. It may well be that what we are proposing is an approach that fits within mixed methodology, bringing together as it does the concept of rich data and quotations from qualitative research and the quantitative approach of aggregating data.

On a practical level, it is important that the discussion of the research methods details the approach taken to producing quotations, so that there is no risk of misrepresenting individuals. How many quotations and from how many individual contributors should be identified for each aggregated quotation used so that the quotations create a rich, comprehensive picture of what a range of participants feel while maintaining clarity and openness over the process.

This research was carried out with people contributing to online discussion boards, rather than any other type of online communication, such as chat rooms. The important consideration is that on discussion boards messages are typed and posted into a durable format. People posting are able to see a record of other posts and answers available to browse or be searched. Chat rooms and other similar forums suggest a more transient online presence (even if not a technically accurate perception), and previous research [44] has found that participants may have different responses. There is therefore no suggestion that these findings are transferable outside of discussion board-type communications.

### Conclusion

The Internet is still a relatively young entity, and people are still developing their understanding of the potential conflicts between the public reality and private use of social media. It does however provide a source of rich research data about the experiences of people living with long-term conditions, among other things. This research found that people who contribute to discussion boards are doing so to share information and help others within that community and are sympathetic to researchers using the information they have shared to further that aim.

Acting ethically as researchers within this new genre is, however, a challenge. Trying to apply rules created for different situations does not provide a solution to how to use the information in health research. Therefore, the solution can be only to create a new set of norms that meet both the needs of researchers for rigor and openness in their research and the wish of posters to contribute, while protecting their personal information. The proposals in this paper, particularly the need to handle data from discussion boards in a new way, are offered as a practical way forward. They are also shared with the research and patient communities to offer a starting point for discussion.

## Multimedia Appendix 1

Questions asked.
