# Supplementary material for: The Conceptual and Practical Ethical Dilemmas of Using Health Discussion Board Posts as Research Data
Source: J Med Internet Res. 2013 Jun 7;15(6):e112. doi: 10.2196/jmir.2435 (PMC3713935; doi:10.2196/jmir.2435)
Supplement: Supplementary file 1 [file jmir_v15i6e112_app1.pdf]

Multimedia Appendix 1

Questions asked

Who do you think reads the information that you post on the board?

Why do you think they read it?

Once you have posted on [message board], how do you hope people will use that post?

What permission do you think I should obtain (if any) before using this information?

Do you think that the length of time since the post was made matters in the need to obtain permission?

Additional questions if respondent says they do think permission is needed

In your answer you say that you think researchers like me should ask for permission. How do you think this should be done?

Do you think the board owner should be able to give permission on behalf of all the board contributors?
